# The guanine exchange factor Gartenzwerg and the small GTPase Arl1 function in the same pathway with Arfaptin during synapse growth

**DOI:** 10.1242/bio.011262

**Published:** 2015-06-26

**Authors:** Leo Chang, Tabita Kreko-Pierce, Benjamin A. Eaton

**Affiliations:** Department of Physiology, Barshop Institute for Longevity and Aging Studies, University of Texas Health Science Center at San Antonio, San Antonio, TX 78229, USA

**Keywords:** Arfaptin, Synapse growth, Golgi apparatus, Small GTPase

## Abstract

The generation of neuronal morphology requires transport vesicles originating from the Golgi apparatus (GA) to deliver specialized components to the axon and dendrites. *Drosophila* Arfaptin is a membrane-binding protein localized to the GA that is required for the growth of the presynaptic nerve terminal. Here we provide biochemical, cellular and genetic evidence that the small GTPase Arl1 and the guanine-nucleotide exchange factor (GEF) Gartenzwerg are required for Arfaptin function at the Golgi during synapse growth. Our data define a new signaling pathway composed of Arfaptin, Arl1, and Garz, required for the generation of normal synapse morphology.

## INTRODUCTION

Polarized cells, such as neurons, require highly specialized secretory pathways to grow and maintain their distinct functional domains, and failure of these processes is associated with neuronal dysfunction and pathology (Fan et al., 2008). Current data support the fundamental and conserved requirement for the Golgi apparatus (GA) during the growth and development of dendritic arbors and presynaptic nerve terminals (Ehlers, 2013; Horton et al., 2005; Mehta et al., 2005; Murthy et al., 2003; Ye et al., 2007; Zhou et al., 2014). The dysfunction of the secretory pathway and altered neuronal morphology are common cellular etiologies in motor neurons during lower motor diseases such as amyotropic lateral sclerosis (ALS). This includes lower motor diseases linked to mutations in the Dctn1 and Dhc genes, which encode components of the dynactin complex. The phenotypic analysis of disease models, as well as human patients, harboring mutations in the components of the dynactin complex suggest that dysfunction of the GA is important etiology of lower motor disease (Chang et al., 2013; Gonatas et al., 2006; Laird et al., 2008). Currently, the role of the dynactin complex at the GA in motor neurons is incompletely described and the link between dynactin complex function and motor disease remains unclear.

*Drosophila* Arfaptin (Arfip), the homologue of mammalian Arfaptin2, is a BAR domain protein that it required for normal growth of the Drosophila NMJ during larval development (Chang et al., 2013). Arfaptins have been implicated in vesicle formation at the GA and in the cell periphery (Mim et al., 2012; Peter et al., 2004). In *Drosophila*, Arfip is enriched in the nervous system where it mediates binding of the dynactin complex to the membranes of the neuronal GA consistent with the established requirement of the dynein motor for normal Golgi function and vesicle transport (Caviston and Holzbaur, 2006; Chang et al., 2013; Fath et al., 1994; Mim et al., 2012; Vaughan, 2005; Vaughan et al., 2002). Thus Arfip represents a central component of a vesicle transport system required for normal growth of the NMJ. Here we extend these results by presenting data supporting that the small GTPase Arl1 and the guanine-exchange factor (GEF) Gartenzwerg (Garz) support Arfip function at the GA during synapse growth. Our data support a model in which Arl1 and Garz work in the same genetic pathway to regulate the recruitment of Arfip to Golgi membranes in the motor neuron.

## RESULTS AND DISCUSSION

### The small GTPase Arl1 regulates Arfip membrane localization in *Drosophila*

**Fig. 1.**
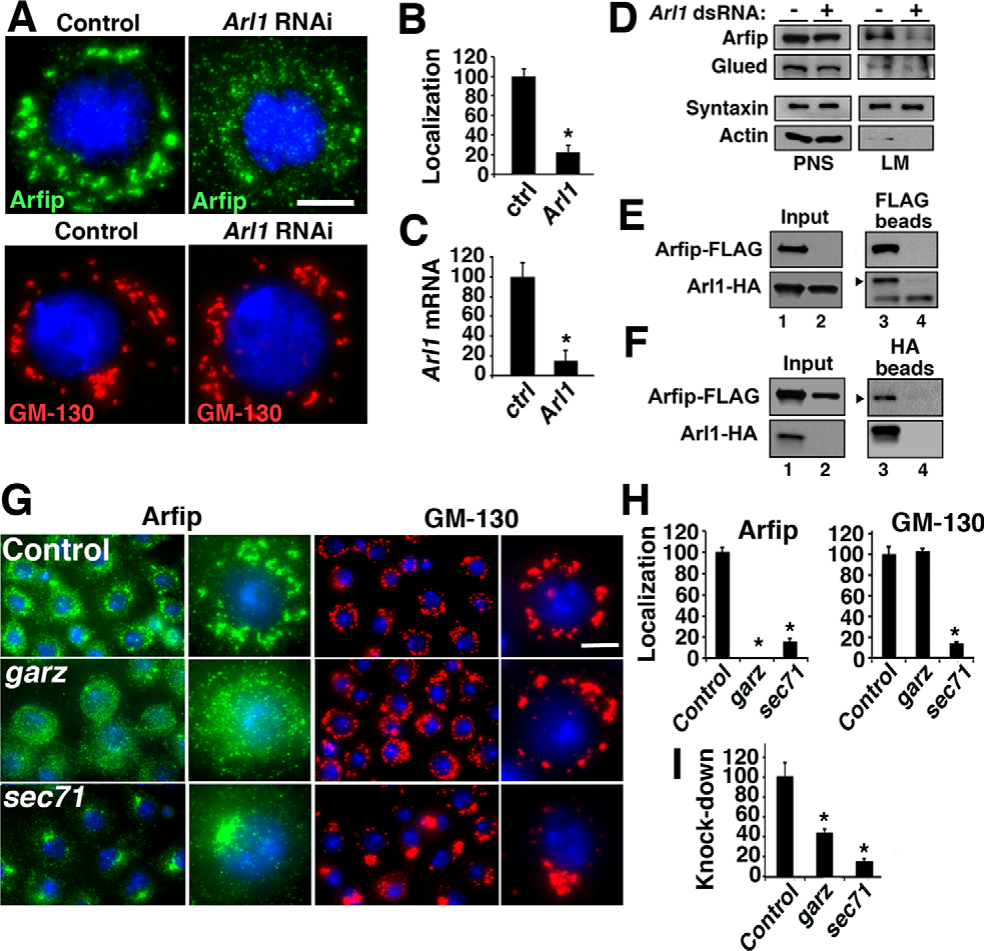
**Arl1 and Gartenzwerg are required for Arfip localization to the Golgi.** (A) Control and *Arl1* dsRNA-treated S2 cells stained with antibodies for either Arfip or GM-130 show that knockdown of *Arl1* disrupts Arfip localization but not GM-130. (B) Graph represents the percent of treated cells with disrupted Arfip staining. (C) Graph represents the effectiveness of *Arl1* RNAi determined using quantitative RT-PCR. Relative expression was determined by the ratio of message to alpha-tubulin normalized to the no dsRNA control. (D) Panel of representative immunoblots of membrane flotation analysis of S2 cells treated with *Arl1* dsRNA or control dsRNA. Syntaxin and actin were markers used to monitor fractionation of light membranes (LM) and soluble proteins (PNS, post-nuclear supernatant), respectively. **(**E,F**)** Immunoblots of co-immunoprecipitation analysis from S2 cells co-expressing FLAG-Arfip and Arl1-HA fusion proteins (E and F; lane 1) incubated with either (E) anti-FLAG or (F) anti-HA antibody-coated beads. Incubation with anti-FLAG beads precipitated Arl1-HA (E; lane 3, arrowhead) and incubation with anti-HA beads precipitated Arfip-FLAG (F; lane 3, arrowhead). Lysate from S2 cells only expressing Arl1-HA (E; lane 2) when incubated with FLAG beads showed no binding of Arl1-HA to anti-FLAG beads (E; lane 4, arrowhead). Similarly, FLAG-Arfip only lysate incubated with anti-HA beads show no binding to anti-HA beads (F; lane 4, arrowhead). Cell lysate of S2 cells co-expressing FLAG-Arfip and Arl1-HA (F; lane 1) or either protein alone (F; lane 2) shows nearly equivalent amount of protein in each co-immunoprecipitation experiment. (G) Immunofluorescence images of fixed S2 cells stained with either anti-Arfip (green) or anti-GM130 (red) antibody. Nuclei are indicated by DAPI staining (blue). The cells were incubated with control dsRNA (GFP) or dsRNA targeting all Arf-like guanine-nucleotide exchange factors (GEFs) in *Drosophila* (see Table 1). (H) Graphs represent the percent of cells with disrupted Arfip (left graph) or GM130 (right graph) staining compared to control. (I) Graphs represent the effectiveness of *garz* and *sec71* RNAi determined using quantitative RT-PCR. Relative expression was determined by the ratio of message to alpha-tubulin normalized to the no dsRNA control. Error bars=s.e.m. *P<0.01; Student's *t*-test (B,C) or ANOVA (H,I). Scale bars =5 μm.

Recent results from mammalian tissue culture cells support that the Arf-like 1 (Arl1) protein regulates the interaction of mammalian Arfaptin1 and Arfaptin2 with the Golgi (Man et al., 2011). Analysis of protein sequence shows that *Drosophila* and human Arl1 share 78% identity in their amino acids, suggestive of well-conserved protein function between species. The *Drosophila* S2 cell provides a simple system to investigate the GA localization of Arfip which we will exploit to identify molecules involved in the regulation of Arfaptin function (Chang et al., 2013). In agreement with the mammalian data, perinuclear distribution of endogenous Arfip in S2 cells was abolished in cells treated with dsRNA for Arl1 (Fig. 1A,B; Chang et al., 2013). In these analyses, we observed an ∼90% reduction in Arl1 mRNA of cells treated with Arl1 dsRNA (Fig. 1C). *Arl1* RNAi had no effect on the localization on GM-130 supporting that Arl1 is not required for general Golgi structure in S2 cells (Burguete et al., 2008; Lu and Hong, 2003; Panic et al., 2003; Setty et al., 2003; Wu et al., 2004). We hypothesized that the effects of *Arl1* RNAi on the Arfip staining pattern was due to reduced Arfip membrane binding and not a reduction in Arfip proteins levels (Chang et al., 2013). Consistent with this hypothesis, cells treated with Arl1 dsRNA did not have a significant reduction in Arfip protein levels in post-nuclear supernatants (Fig. 1D, PNS) but did show a significant reduction of membrane-bound Arfip and Glued (Fig. 1D, LM; Chang et al., 2013).

**Abbreviations**

NMJ: neuromuscular junction
GA: Golgi apparatus
GEF: guanine exchange factor
MTOC: microtubule organizing center

The results above predict that Arl1 and Arfip should physically bind to each other. Co-immunoprecipitation (Co-IP) experiments using cell lysates prepared from S2 cells co-expressing HA-tagged Arl1 and FLAG-tagged Arfip incubated with anti-FLAG antibody coated beads finds that the anti-FLAG coated beads can successfully co-precipitate both the Arfip-FLAG target and Arl1-HA (arrowhead; Fig. 1E, lane 3). Cell lysate made from cells only expressing Arl1-HA (Fig. 1E, lane 2) failed to immunoprecipitate Arl1-HA when incubated with anti-FLAG antibody coated beads (Fig. 1E, lane 4) demonstrating the requirement of Arfip-FLAG for the co-immunoprecipitation of Arl1-HA. Similarly, cell lysate prepared from S2 cells co-expressing HA-tagged Arl1 and FLAG-tagged Arfip incubated with anti-HA antibody successfully co-immunoprecipitated Arl1-HA and Arfip-FLAG (arrowhead; Fig. 1F, lane 3). Control cell lysate prepared form S2 cells only expressing FLAG-tagged Arfip (Fig. 1F, lane 2) incubated with anti-HA antibody coated beads failed to immunoprecipitate the FLAG-tagged Arfip (Fig. 1F, lane 4). These results demonstrate that Arl1 and Arfip are contained within the same biochemical complex and support the assertion that Arl1 regulates the binding and localization of Arfip to Golgi membranes in *Drosophila* neurons (Man et al., 2011).

### RNAi screen for guanine-exchange factors required for Arfip localization identifies Gartenzwerg and Sec71

The cycling between active GTP-bound forms and inactive GDP-bound forms regulates the function of small GTPases, such as Arl1. The activation of small GTPases is largely controlled by guanine-exchange factors (GEFs) that serve to exchange a GDP for a GTP. To identify the GEF responsible for the localization of Arfip to the Golgi, we performed an RNAi screen in S2 cells with dsRNAs targeting all of the identified genes encoding GEFs in *Drosophila* to identify dsRNAs that altered the normal localization of endogenous Arfip. We expected that RNAi knockdown of the appropriate GEF would phenocopy of the effects of *Arl1* RNAi (Fig. 1A). An RNAi screen of all putative GEFs found that RNAi knockdown of *gartenzwerg* (*garz*) and *Sec71* resulted in the disruption of normal Arfip localization, although the effects of *Sec71* RNAi on Arfip localization were strikingly different than the effects of *garz* RNAi (Fig. 1G,H; Table 1). Specifically, RNAi of *Sec71* resulted in the redistribution of both Arfip and GM-130 to a juxtanuclear localization within the cell (Fig. 1G-I). This is consistent with a broader role of Sec71 for the regulation of normal Golgi structure. In contrast to the effects of *Sec71* knockdown, RNAi knockdown of *garz* phenocopied the effect of *Arl1* RNAi on both Arfip and GM-130 localization (Fig. 1G-I). These data supports that Garz is required for Arfip localization to the Golgi in *Drosophila*.

**Table 1.**
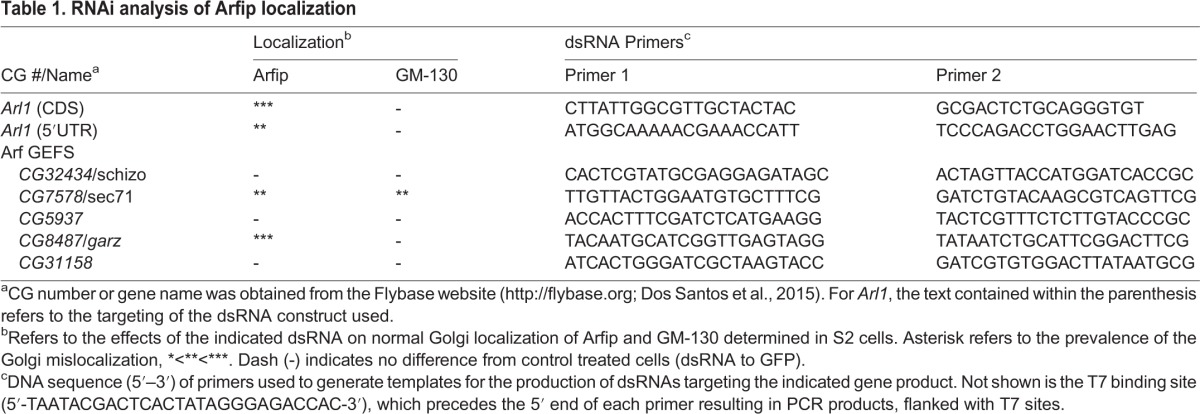
**RNAi analysis of Arfip localization**

### Co-localization of Arl1 and Arfip in cells and neurons

**Fig. 2.**
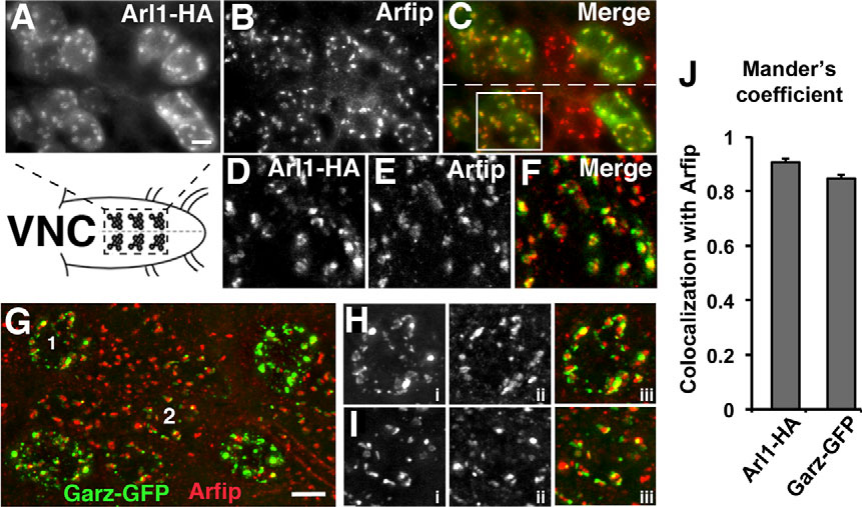
**Colocalization of Arl1, Garz, and Arfip at the Golgi in motor neurons.** (A-F) Images of indicated portion ventral nerve cord harboring motor neuron soma from a 3rd instar larvae expressing Arl1-HA using the *C155-Gal4* line in the motor neurons co-stained for (A) Arl1-HA using the anti-HA antibody and for (B) Arfip using the anti-Arfip antibody. The merged image of panels A and B is shown in panel C. Dashed line indicates the midline. (D-F) A deconvolved image of the boxed area in C is shown demonstrating the high degree of colocalization of Arl1-HA (D; green in F) and Arfip (E; red in F) in the motor neuron soma. (G) Deconvolved immunofluorescent image of motor neuron soma in larvae expressing Garz-GFP in motor neurons using the *OK6-Gal4* line co-stained with anti-GFP (green) and anti-Arfip (red). (H,I) Higher magnification images of soma 1 (H) and soma 2 (I) indicated in G. Panels i are Garz-GFP, panels ii are Arfip, and panels iii are the merged image. (J) Graphs represent the average Mander's coefficient from neuronal cell bodies comparing Arl1-HA with endogenous Arfip (left bar) or Garz-GFP with endogenous Arfip (right bar). *n*=12–14 soma from 3–5 VNCs. Scale bars=10 μm.

Our model is that Garz and Arl1 represent components of a GTPase signaling system required for normal Arfip function in the motor neuron. This model predicts that these proteins should colocalize in neurons (Chang et al., 2013). Ventral nerve cords (VNCs) from larvae expressing transgenic Arl1-HA using the panneuronal *C155-Gal4* driver were fixed and processed for immunofluorescent microscopy using antibodies against endogenous Arfip and HA. Microscopic 3D imaging of motor neurons reveals that Arl1-HA co-localization with endogenous Arfip in neurons (Fig. 2A-F), similar to what we observe in S2 cells. We further confirmed these data by performing voxel by voxel co-localization analysis on 3D images to generate a Mander's Overlap Coefficient (MOC), which is a quantitative measure of co-localization (Chang et al., 2013). These analyses resulted in an average MOC value consistent with high amounts of co-localization between Arl1 and Arfip (1=perfect colocalization) (Fig. 2J). These localization data are similar to the colocalization of Arl1 and Arfaptin2 in mammalian cells (Man et al., 2011). To investigate the colocalization of Garz with Arfip we employed Garz-GFP expressed in motor neurons using the OK6-Gal4 driver and observed a similar co-localization with endogenous Arfip in the soma of the neuron (Fig. 2G-I). The Mander's Overlap Coefficients for Arfip and Garz-GFP in these analyses was consistent with significant colocalization of Arfip with Garz-GFP within the soma of the motor neurons (Fig. 2J). These data demonstrate that both Arl1 and Garz colocalize with Arfip at the GA in motor neurons.

### Arl1, Garz, and Arfip function in the same genetic pathway during synapse growth

**Fig. 3.**
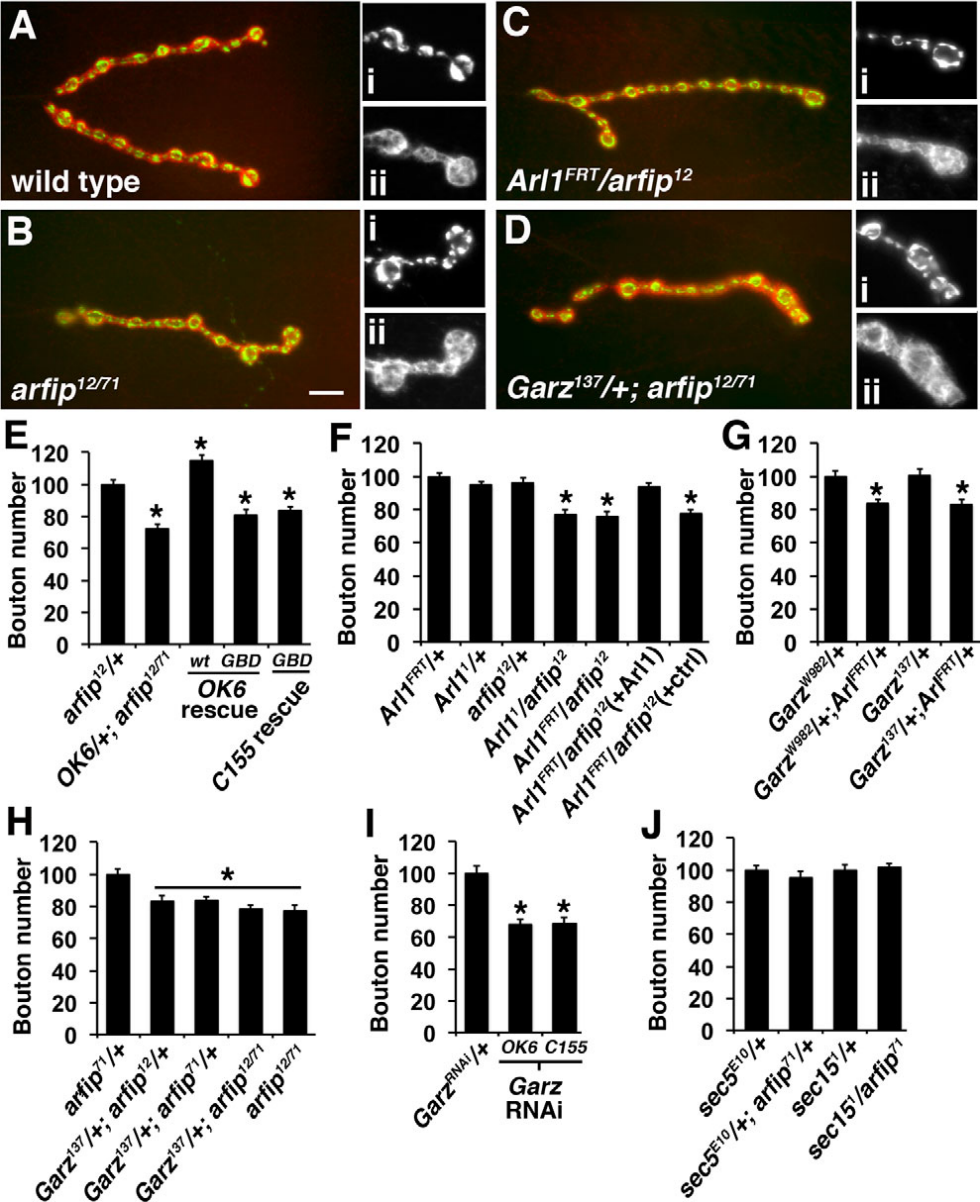
**Arl1, Garz, and Arfip function in the same genetic pathway during synapse growth.** (A-D) Immunofluorescent images of the neuromuscular junctions (NMJs) formed on muscle 4 from larvae of the indicated genotypes co-stained for presynaptic VGluT (green; insets i) and postsynaptic Discs-large (Dlg) (red; insets ii). Insets are higher magnification images of the terminal boutons of the synapse shown. Scale bar=10 μm. (E-I) Graphs represent the average number of boutons at the muscle 4 NMJs normalized to control values. (E) The expression of wild type Arfip (*wt*) but not a GTPase-binding mutant Arfip (*GBD*) rescued synapse growth defects in *arfip^12/71^* mutant neurons using either the *Ok6-Gal4* (Ok6 rescue) or C155-Gal4 (*C155 rescue*). (F) Transheterozygotic genotypes consisting of *Arl1^1^* and *arfip^12^*, *Arl1^FRT^* and *arfip^12^* have reduced synapse growth compared to heterozygotic *Arl1* controls (*Arl1^1^*/+ and *Arl1^FRT^*/+). Expression of the pUAS-Arl1-HA transgene in the *Arl1^FRT^/arfip^12^* heterozygotic background using the *OK6-Gal4* driver [*Arl1^FRT^/arfip^12^* (+Arl1)] restores synapse growth to control levels compared to the control background harboring only the OK6-Gal4 driver [*Arl1^FRT^/arfip^12^* (+ctrl)]. (G) Transheterozygotic genotypes consisting of *Arl1^FRT^* and *garz^W982^* or of *Arl1^FRT^* and *garz^137^* have reduced synapse growth compared to heterozygotic *garz* controls (*garz^W982^*/+ and *garz^137^*/+). (H) Transheterozygotic genotypes containing heterozygotic mutations in *arfip* (*arfip^12^* or *arfip^71^*) and *garz^137^* have reduced synapse growth compared to *arfip*/+. (I) The expression of a UAS construct encoding a dsRNA to *garz* (*UAS-garz^RNAi^*) in motor neurons using either the *Ok6-Gal4* motor neuron driver or the *C155-Gal4* panneuronal driver results in reduced synapse growth. (J) Transheterozygotic genotypes between mutations in *arfip^71^* and the exocyst genes *sec5^E10^* or *sec15^1^* have no effect on synapse growth. Error bars=s.e.m. *P<0.05; significant difference from controls by ANOVA.

All genetic mutations used in the following studies are strong loss of function/null alleles (see Materials and Methods). We find that neuronal overexpression of an Arfip molecule with a mutated GTPase binding domain (GBD) fails to restore synapse growth in *arfip* mutants compared to the neuronal overexpression of wild type Arfip, which we have found results in neuronal overgrowth (Fig. 3E) (Chang et al., 2013). We confirmed this result using two independent Gal4 driver lines, the motor neuron-specific *OK6-Gal4*, and the panneuronal *C155-Gal4* (Sanyal, 2009). These results demonstrate a role for GTPase signaling during Arfip-dependent synapse growth. These genetic analyses were extended to investigate if Garz and Arl1 regulate Arfip activity during synapse growth. Because null mutants in both *Arl1* and *garz* are embryonic lethal (Armbruster and Luschnig, 2012; Tamkun et al., 1991; Wang et al., 2012) we have used trans-heterozygotic backgrounds to investigate whether *Arl1*, *garz*, and *arfip* function in the same genetic pathway during synapse growth. All alleles used in the subsequent studies have been previously characterized as representing amorphic alleles (see Methods). We observe that trans-heterozygotic combinations of *Arl1^FRT^*,* Arl1^1^*,** and *arfip^12^* mutations have reduced synapse growth as determined by counting the number of boutons at the neuromuscular junction (NMJ) formed on 3rd Instar larval muscle 4 (Fig. 3A-F). Expression of Arl1-HA under control of the OK6-Gal4 in the *Arl1^FRT^/arfip^12^* trans-heterozygote background restored bouton numbers to control values (Fig. 3F). These effects on synapse growth are consistent with *arfip*, and *Arl1* functioning within the same genetic pathway during synapse growth. Reduced synapse growth phenotype is also observed in trans-heterozygotic combinations of *Arl1^FRT^* with both *garz^137^* and *garz^W982^* (Fig. 3G). A similar genetic interaction is observed between either *arfip^12^*or *arfip^71^* mutations and the *garz^137^* mutation (Fig. 3D,H). Further, removal of a single copy of *garz* in an *arfip* mutant background does not result in an enhancement of the synapse growth defect observed in *arfip* mutants supporting that Garz functions in the same pathway with Arfip during synapse growth (Fig. 3D,G). These results demonstrate that *garz*, *Arl1*, and *arfip* function in the same genetic pathway within the neuron during synapse growth. In support of this model, neuron specific RNAi of *garz* results in a reduction in synapse growth (Fig. 3H).

Our data here demonstrate that Arl1 is required for Arfip membrane binding, Arfip Golgi localization, and synapse growth. Because of the proposed role of BAR domain proteins during vesicle biogenesis (Campelo and Malhotra, 2012), we propose that Arfip binding to GTP-bound Arl1 facilitates the biogenesis of transport vesicles required for the growth of the nerve terminal. Consistent with this model is the observation that *garz* RNAi leads to both the loss of Arfip staining from the GA and the reduction in synapse growth at the NMJ. Further, we observe a reduction of synapse growth in trans-heterozygotic combinations between *garz* mutations and both *Arl1* and *arfip* mutations. These data are consistent with reduction of Garz function resulting in decreased levels of Arl1-GTP and reduced Arfip function during synapse growth.

Previous data has shown that the exocyst complex, known to function during vesicle formation at the Golgi, is required for the delivery of synaptic proteins, such as synaptotagmin, to the nerve terminal (Murthy et al., 2003). The synapse growth pathway defined by Arfip, Arl1, and Garz likely functions independent of the exocyst complex as no synergistic effects were observed in transheterozygotic combinations between *arfip* mutations and mutations in the *sec5* or *sec15* genes (Fig. 3I). These data support that *arfip* represents a distinct pathway originating from the GA that is also required for normal synapse growth. Interestingly, there is no functional consequence of *arfip* mutations on synaptic vesicle release as determined quantal analysis of evoked release which is similar to the phenotype of sec5 mutants (Table 2) (Murthy et al., 2003). Our data further support that membrane trafficking required for normal neuronal growth and development are distinct from the pathways supporting neurotransmitter release, which in part has been attributed to a distinct class of Golgi-derived vesicle referred to as Piccolo-Bassoon transport vesicles (PTVs) (Shapira et al., 2003). The utilization of GTPase signaling systems could provide the biochemical diversity to generate the distinct vesicle classes required for the growth and function of the neuron.

**Table 2.**
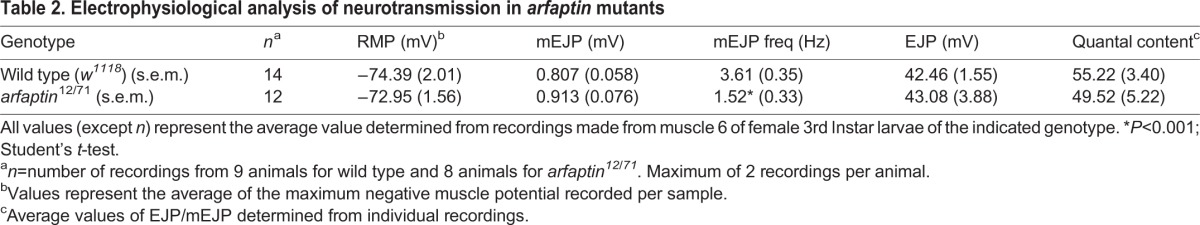
**Electrophysiological analysis of neurotransmission in *arfaptin* mutants**

## MATERIALS AND METHODS

### Fly stocks

All fly stocks were maintained on a standard fly food (Bloomington Stock Center) at 25°C and 50% humidity. The following *gartenzwerg (garz)* stocks were used in this study: *garz^137^* (small deletion generated by transposon excision), *garz^W982^* (EMS-generated point mutation) and *UAS-GFP-garz* (Armbruster and Luschnig, 2012; Wang et al., 2012). Both *garz* mutations are strong loss of function/null alleles. The *pUAS-garz^RNAi^* line was obtained from the Bloomington Stock Center (stock# 31232). The *Arl1^1^*allele (EMS-generated point mutation) is a strong loss of function/null allele (Tamkun et al., 1991). The *Arl1^FRT^* deletion was made using Flippase-mediated recombination following the protocol found at the DrosDel website (http://www.drosdel.org.uk/) using the following P-element insertions: P{XP}d07562 and PBac{WH}f02582. The resulting deletion completely removes the Arl1 gene as well as the following neighboring genes: CG10516, CG17029, CG6025 (Arl1), CG5942, CG17028, CG5949, CG17026, CG17027. The *pUAS-Arl1-HA* gene was generated by PCR cloning from the GM20805 cDNA (Drosophila Genomics Resource Center, Bloomington IN). To generate the *pUAS-arfip^GBD^-HA* vector, a *pUASt-Arfip^WT^-HA* vector was modified by inserting mutations H170A/Q172A/H173A into the GTPase binding pocket to abolish binding of small GTPases without altering membrane binding (Tarricone et al., 2001; TOP Gene Technologies, Montreal, Quebec, Canada). All transgenic lines were generated by microinjection into *w^1118^* (Rainbow Transgenics, Camarillo, CA).

### dsRNA interference in S2 cells

S2 cells were incubated with 10 μg/ml of dsRNA for the respective targets for five days and the media were changed daily. At the end of the RNAi treatment, cells were fixed and processed for Arfip localization using a rabbit anti-Arfip antibody. The effectiveness of the RNAi on Arfip and GM-130 localization was determined by quantifying the number of cells with correct localizaion from 10 randomly chosen fields of view (∼500 cells total). To verify knock-down, real-time PCR using Syb-R Green (Stratagene) with gene specific primers (Table 1).

### Cell transfection and co-immunoprecipitations

A polyclonal S2 cell line that constitutively expresses Gal4 was used for all experiments and maintained as previously described (Chang et al., 2013). Transient transfection of S2 cells was performed using a calcium phosphate based method as described in the *Drosophila* Expression System Kits manual (Invitrogen). For co-immunoprecipitation (co-IP) two 80% confluent 10 cm dishes of cells co-transfected with appropriate plasmids were homogenized in cold IP buffer [IPB; 50 mM Tris (7.4), 50 mM NaCl, 1% TX-100, 1 mM MgCl_2_, and protease inhibitors]. Cell lysate was incubated with anti-FLAG or anti-HA coated beads (Sigma-Aldrich, St. Louis, MO USA) and samples processed as previously described (Chang et al., 2013). The following primary antibodies were used for all immunoblot analysis: rabbit anti-Arfip (1:1000; Chang et al., 2013), mouse anti-HA (1:1000; US Biologicals, Salem, MA USA), rabbit anti-FLAG (1:1000; Sigma-Aldrich). All secondary antibodies were used at a 1:10,000 dilution. Blots were exposed to autoradiographic film and visualized using the ECL Chemiluminesence kit (Amersham, Piscataway, NJ USA).

### Membrane flotation

For flotation analysis, 3–4 10 cm dishes of Arfip dsRNA-treated cells and non-dsRNA-treated control cells were homogenized in homogenization buffer (8% sucrose and 3 mM imidazole, pH 7.4). A post nuclear supernatant was obtained by centrifuging at 1000×***g*** for 7 min in standard tabletop microcentrifuge. The resulting PNS was brought to 40% sucrose, bottom loaded, and overlaid with two cushions of 35 and 8% sucrose. The gradient was centrifuged at 28,000 rpm for 1 h in a TH641 rotor. Light membranous organelles (LM) and soluble proteins were recovered and equal amounts of protein from each fraction were analyzed by SDS-PAGE and immunoblotting using the following antibodies: rabbit anti-Arfip (1:1000; Chang et al., 2013), mouse anti-syntaxin (1:500; Developmental Studies Hybridoma Bank, Iowa City, IA USA), mouse anti-actin (1:500, Sigma-Aldrich).

### Microscopy

All fixed images were captured with an Orca-2 back-cooled CCD camera attached to a Zeiss Axiovert 200M. Samples were fixed for 10 min using 4% paraformaldehyde in 1× PBS followed by permeabilization with 1× PBS/0.1% TX-100. The following primary antibodies were used in these studies: rabbit anti-GM-130 (1:500; Cell Signaling Technology, Danvers, MA USA), mouse anti-Discs-large (1:400; Developmental Studies Hybridoma Bank), rabbit anti-VGlut (1:10,000; Chang et al., 2013), rabbit anti-Arfip (1:500; Chang et al., 2013), mouse anti-HA (1:500; US Biologicals), rabbit anti-FLAG (1:1000; Sigma-Aldrich), and mouse anti-FLAG (1:500; Sigma-Aldrich). All secondary antibodies were used at 1:400 dilution (Life Technologies, Grand Island, NY USA). For Mander's Overlap Coefficient (MOC) analysis z-stack images of S2 cells or larval motor neurons were generated at 160× and deconvolved using a constrained iterative algorithm that utilizes a theoretical point-spread function as previously described (Chang et al., 2013). The deconvolution and colocalization analyses are built in features of the Slidebook software controls our imaging system (Intelligent Imaging Inc., Denver, CO USA). For analysis of synaptic growth, fixed 3rd Instar larval NMJs from indicated genotypes were processed for immunofluorescent microscopy with antibodies to *Drosophila* VGluT and Discs-large. The number of boutons was determined at the NMJ on muscle 4 of segment A3 of the larvae. Bouton counts were normalized to muscle size and control genotype.

### Electrophysiology

Recordings were performed under standard conditions as described previously (Eaton et al., 2002). Briefly, recordings were performed on muscle 6 of abdominal segment 4 in HL3 (Ca^2+^ – 2 mM, Mg^2+^ – 20 mM). Only muscles that had a resting membrane potential <−60 mV were used for data analysis. Data was acquired with a PowerLab 4/30 (ADInstruments) and LabChart7 Pro (ADInstruments). EJPs and mEJPs were measured with Mini Analysis (Synaptosoft). For each muscle 50 mEJPs and 10 compound EJPs were measured to calculate the average values. Unadjusted quantal content is reported here.

### Statistical analysis

Statistical significance was determined by a Student's *t*-test for pair-wise comparison, and comparison of more than two conditions utilized ANOVA with a Bonferroni correction. All values are presented as an average with the error bars representing s.e.m. *P* values less that 0.05 were considered significant.
